# USING DIGITAL TECHNOLOGIES TO IMPROVE READMISSION AND ADHERENCE AMONG OLDER ADULTS WITH HEART FAILURE

**DOI:** 10.1093/geroni/igac059.3140

**Published:** 2022-12-20

**Authors:** Melissa O’Connor, Nilmini Wickramasinghe, Vijay Gehlot, Elliot Sloane, Suresh Chalasani, Ramona Wallace, Patricia G Wisk, Stefan Gravenstein

**Affiliations:** Villanova University, M. Louise Fitzpatrick College of Nursing, Villanova, Pennsylvania, United States; Swinburne University of Technology & Epworth HealthCare, Hawthorne, Victoria, Australia; Villanova University, Villanova, Pennsylvania, United States; Villanova University, Villanova, Pennsylvania, United States; University of Wisconsin-Parkside, Kenosha, Wisconsin, United States; Western Michigan University, Kalamazoo, Michigan, United States; University of Illinois at Chicago, Holland, Michigan, United States; Brown University, Providence, Rhode Island, United States

## Abstract

Heart failure (HF) affects approximately 26 million people globally resulting in morbidity, mortality, and rehospitalization. With 30-day rehospitalization rates ranging from 19% to 25%, HF is burdensome for patients and challenging for clinicians to manage. HF costs in the U.S. exceeded $30 billion in 2012, projected to exceed $70 billion by 2030, and produce estimated social care costs of 12 billion in 2020. Therefore, reducing HF readmissions should be a global priority. A technology-based platform may assist in the goal of reducing readmissions. This platform may accomplish this goal by providing several digital tools that can assist the clinician in the care process, while simultaneously enabling patients with self-care through more effective communication in all care settings. Digital twins (DT) and the nursing Avatar (NA) are two such tools. The DT is a virtual, real-time digital representation of a physical object/process. It originated from NASA to improve physical model simulation of spacecraft in 2010 (Wickramasinghe et al., 2021). It is possible to achieve similar benefits in healthcare by developing suitable DT of patients in specific clinical contexts (Wickramasinghe et al., 2021; Lui et al., 2019). So to, the NA was studied by Bickmore, et al (2209) and found ultimately to be the preferred discharge communication method. As such, we proffer the development of digital twins and NA for HF patients to support better provider decision and patient communication. This will result in HF care being more precise, consistent, reproducible, and personalized resulting in improved diagnosis, treatment, and care outcomes.

